# Efficacy and safety of artemisinin-based combination therapy for uncomplicated *Plasmodium falciparum* malaria in Sudan: a systematic review and meta-analysis

**DOI:** 10.1186/s12936-018-2265-x

**Published:** 2018-03-13

**Authors:** Ishag Adam, Yassin Ibrahim, Gasim I. Gasim

**Affiliations:** 1 Faculty of Medicine, University of Khartoum, P.O. Box 102, Khartoum, Sudan; 2 Faculty of Medicine, University of Tabuk, P.O. Box 741, Tabuk, Kingdom of Saudi Arabia; 3 Faculty of Medicine, Alneelain University, Khartoum, Sudan

**Keywords:** Malaria, Treatment, Outcome, Efficacy, *Plasmodium*, Systematic review, Meta-analysis, Sudan

## Abstract

**Background:**

Malaria is a major public health problem in endemic countries including Sudan, where about 75% of populations are at risk. Due to widespread of chloroquine-resistant strains of *Plasmodium falciparum*, artemisinin-based combination therapy (ACT) is currently treatment of choice for malaria in the vast majority of malaria-endemic countries. This systematic review and meta-analysis is performed to obtain an overall stronger evidence of the outcomes of ACT in the treatment of uncomplicated falciparum malaria from the existing literature in Sudan.

**Methods:**

The preferred reporting items for systematic review and meta-analysis statement were used to select studies to be included in this review. A computerized systematic strategy was adopted to search articles from PubMed, Google Scholar and Science Direct databases. Unpublished materials were also included. Open Meta-Analyst software was used to perform the meta-analysis. Random effects model was used to combine the included studies and the heterogeneity of studies was assessed using Cochrane Q and I^2^ (χ^2^ = 73.05, *df* (19), *P* < 0.001 and I^2^ = 73.99).

**Results:**

Twenty studies fulfilled the inclusion criteria (ACT in the treatment of uncomplicated falciparum malaria) and were included in the final analysis with a total number of 4070 participants. Malaria treatment outcome was assessed using World Health Organization guidelines. Adequate clinical and parasitological response was used to assess treatment success at the 28th day. Treatment success of all combined studies was 98% [(95% CI 97.2–98.8%), *P* < 0.001]. Treatment success was higher in malaria patients treated with artemether + lumefantrine (AL) than patients treated with artesunate + sulfadoxine–pyrimethamine (AS + SP) (98.9% (95% CI 98.4–99.4%) vs 97.1% (95% CI 95.5–98.6%), *P* < 0.001). Eleven studies reported adverse drug reactions (ADRs) to ACT (184 participants out of 3957 (4.65%). The ADRs were mild and resolved spontaneously. There was no severe ADRs or deaths.

**Conclusion:**

Based on this review, the overall malaria treatment success was high (98%). AL regimen showed higher efficacy compared to AS + SP. The overall regimens were associated with mild low rates ADRs.

**Electronic supplementary material:**

The online version of this article (10.1186/s12936-018-2265-x) contains supplementary material, which is available to authorized users.

## Background

Despite effective control measures, malaria remains a major public health concern, with 212 million new malaria cases and an estimated 429,000 malaria-related deaths globally, with sub-Saharan Africa accounting for approximately 90% of malaria cases and deaths [1]. Early diagnosis and timely treatment of malaria with an effective drug is an important strategy to control the disease [2]. However, this goal is hampered by the emergence of anti-malarial drug resistance which is one of the main challenges to controlling and eliminating malaria [3].

Owing to widespread chloroquine-resistant *Plasmodium falciparum* strains, artemisinin-based combination therapy (ACT) is currently the adopted treatment of uncomplicated falciparum malaria in most endemic countries [4].

Malaria constitutes a major public health problem in Sudan. According to the 2015 annual estimate, there were 586,827 confirmed cases and 3500 deaths due to malaria, and malaria represents 8.7% of total outpatient attendance and 12.2% of hospital admissions [5]. Sudan changed its policy for treatment of uncomplicated falciparum malaria from chloroquine to ACT in 2004, since when artesunate + sulfadoxine–pyrimethamine (AS + SP) and artemether–lumefantrine (AL) is, respectively, the first and second-line treatment for uncomplicated falciparum malaria [6].

Although there are several studies that were conducted to assess the efficacy of malaria treatment agents yielding different success rates in Sudan, there has been no systematic review and/or meta-analysis conducted to obtain strong evidence about the outcome of malaria treatment. This systematic review and meta-analysis is performed to obtain evidence on the efficacy and safety of ACT in the treatment of uncomplicated falciparum malaria in Sudan.

## Methods

### Searching strategies

The preferred reporting items for systematic review and meta-analysis (PRISMA) statement were used to select studies to be included in this review. A computerized systematic strategy was adopted to search articles from PubMed, Google Scholar, and Science Direct databases [7]. Both interventional and observational studies were retrieved to be included in the review using the terms: Sudan AND malaria AND (treatment OR management) AND (artemether–lumefantrine OR artesunate OR chloroquine OR mefloquine OR primaquine OR pyrimethamine) OR resistance. Only studies that were conducted in Sudan were included. All research articles published and unpublished before 20 October 2017 in English language were included in this review. Retrieved studies that were included or excluded, in addition to unpublished articles, are shown in Additional file 1. In addition to published data from databases, unpublished findings from the National Malaria Control Programme, Ministry of Health, Sudan were also included (Additional file 1).

### Inclusion criteria

Original articles of studies that investigated ACT in the treatment of uncomplicated falciparum malaria, written in English and conducted in Sudan were included in this systematic review and meta-analysis. PICOS format was used to select and include studies (Additional file 1). The primary objective of this review was the efficacy of ACT measured as treatment success at day 28 for uncomplicated malaria caused by *P. falciparum*, while the frequency of adverse drug reactions (ADRs) was the secondary objective. ADRs were defined as ‘signs and symptoms that first occurred or became more severe after treatment was started’ or ‘as a sign, symptom, or abnormal laboratory value not present on day 0, but which occurred during follow up, or was present on day 0 but became worse during follow up’. Serious adverse events were defined according to International Conference on Harmonization (ICH) guidelines. Studies included in this review are shown in Additional file 1.

### Exclusion criteria

Studies that used chloroquine, artemisinin monotherapy and those that assessed malaria treatment outcomes at times less than 28 days were excluded from this systematic review and meta-analysis. Studies that were excluded from this review are shown in Additional file 1.

### Study search

ENDNOTE software version X8 (Thomson Reuters, USA) was used to import the research articles from the electronic databases and duplicates were removed. Two reviewers (IA and YI) independently screened titles and abstracts of retrieved articles and identified potentially eligible studies. In case of discrepancy between the two reviewers, a third reviewer (GIG) made the decision. Before the start of data extraction, full-length articles of the selected studies were read to confirm that they fulfilled the inclusion criteria.

### Methodological quality assessment

The quality of the reviewed studies was assessed through sensitivity analysis which classified the included studies into high quality and low quality according to modified Jadad scale for randomized controlled trials (RCTs) [8] and the strengthening the reporting of observational studies in epidemiology (STROBE) statement for observational studies [9]. Modified Jadad scale assesses the quality of a trial with the range from 0 to 8 (randomization and its appropriate use, blinding and its appropriate use, withdrawals and dropouts, description of inclusion and exclusion criteria, assessment of adverse effects, and description of statistical analysis). The score for the modified Jadad scale ranges from a minimum of zero, meaning low quality to a maximum of eight, meaning high quality. Score range of 0–3 represents low or poor quality and score ranges of 4–8 represents good to excellent quality. The observational studies were categorized as low quality with a score under 75% of the STROBE checklist and high quality with a score over 75% of the STROBE checklist. The reviewers independently assessed the quality of the methodology of included studies. All the 20 interventional studies that were included in this review were assessed according to the modified Jadad scale and were found to have high quality (Additional file 1).

### Data extraction

Data related to the author/s of the article, year the studies were conducted, geographic location of the study area, duration of the study, ages and genders of participants, and the type of study design (observational vs interventional) were extracted first from each article. Data regarding the types of malaria treatment agents, treatment duration, treatment outcome measures (including treatment success rates, treatment failure rates, and case fatality rates), and ADRs were extracted and included in the systematic review and meta-analysis.

### Measurement of treatment success

Adequate clinical and parasitological response (ACPR) was used as an indicator for treatment success. ACPR was defined as absence of parasitaemia by the end of treatment (day 28) irrespective of axillary temperature without previously meeting any of the criteria of early treatment failure or late clinical failure or late parasitological failure [10–12]. The outcomes of all the studies included in this review were assessed and analysed at the 28th day of treatment. The treatment success was defined based on PCR genotyping according to current World Health Organization (WHO) recommendation.

### Publication bias

Publication bias was assessed using funnel plot with the sample size of each study plotted against its effect size (Additional file 2).

### Data analysis and heterogeneity assessment

OpenMeta Analyst software for Windows [13, 14] was used to perform all the meta-analyses of malaria treatment efficacy. The heterogeneity of the included studies was evaluated using Cochrane Q and the I^2^. Cochrane Q with *P* < 0.10 and I^2^ > 50 was taken as standard to indicate the presence of heterogeneity of the included studies [15]. Based on the analysis, Cochrane Q and I^2^ indicated that the included studies were heterogeneous (χ^2^ = 73.05, *df* (19), *P* < 0.001 and I^2^ = 73.99). The method of random effects model was used to combine the included studies. A sub-group analysis was performed by comparing the different anti-malarial treatment regimens. ADR rates were calculated and compared for the different anti-malarial medications.

### Ethical considerations

PRISMA guideline recommendations were used and strictly followed to carry out this systematic review and meta-analysis. Since this is a systematic review and meta-analysis, ethical approval is not recommended and was not required.

## Results

### Study selection process

**Fig. 1:**
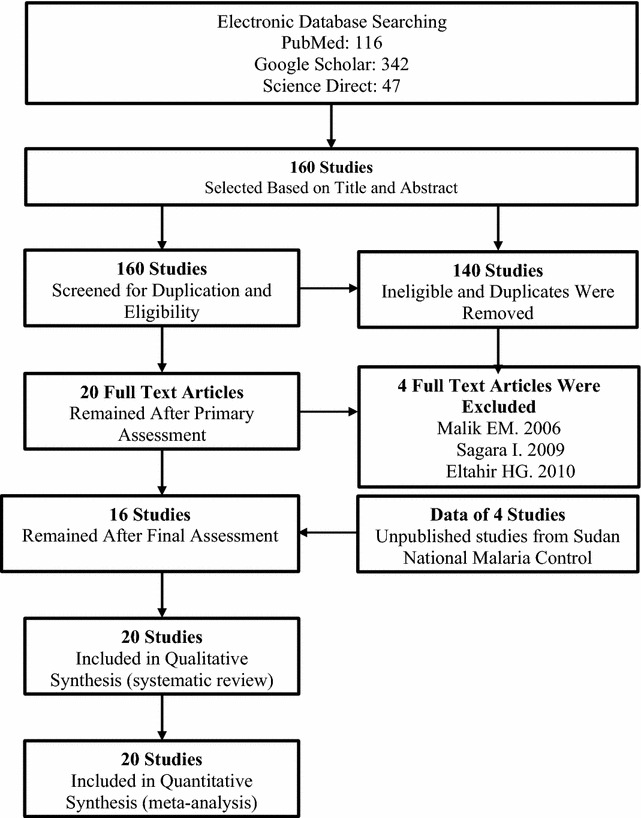
Flow diagram showing the number of articles identified in the systematic review and meta-analysis on anti-malarial treatment outcomes in Sudan

A computerized systematic strategy was adopted to arch articles from PubMed, Google Scholar, and Science Direct databases (Fig. 1).

### Qualitative synthesis

The search of available literature up to 20 October 2017 identified 20 titles relevant to the review topic [6, 16–34], of which 15 were RCTs [16–30], 4 clinical trials without randomization [31–34], and one descriptive case study. Data from 4 unpublished studies that were conducted by the Sudan National Malaria Control Programme (pers. comm.) were also included in this review. Twenty out of the retrieved 24 studies satisfied the inclusion criteria and were included in this systematic review and meta-analysis with a total sample size of 4070 participants that ranged from 30 patients [19] to 1463 patients [23]. Treatment outcomes in all studies were assessed using clinical and parasitological criteria according to WHO guidelines [10–12, 35].

### Quantitative synthesis

**Fig. 2:**
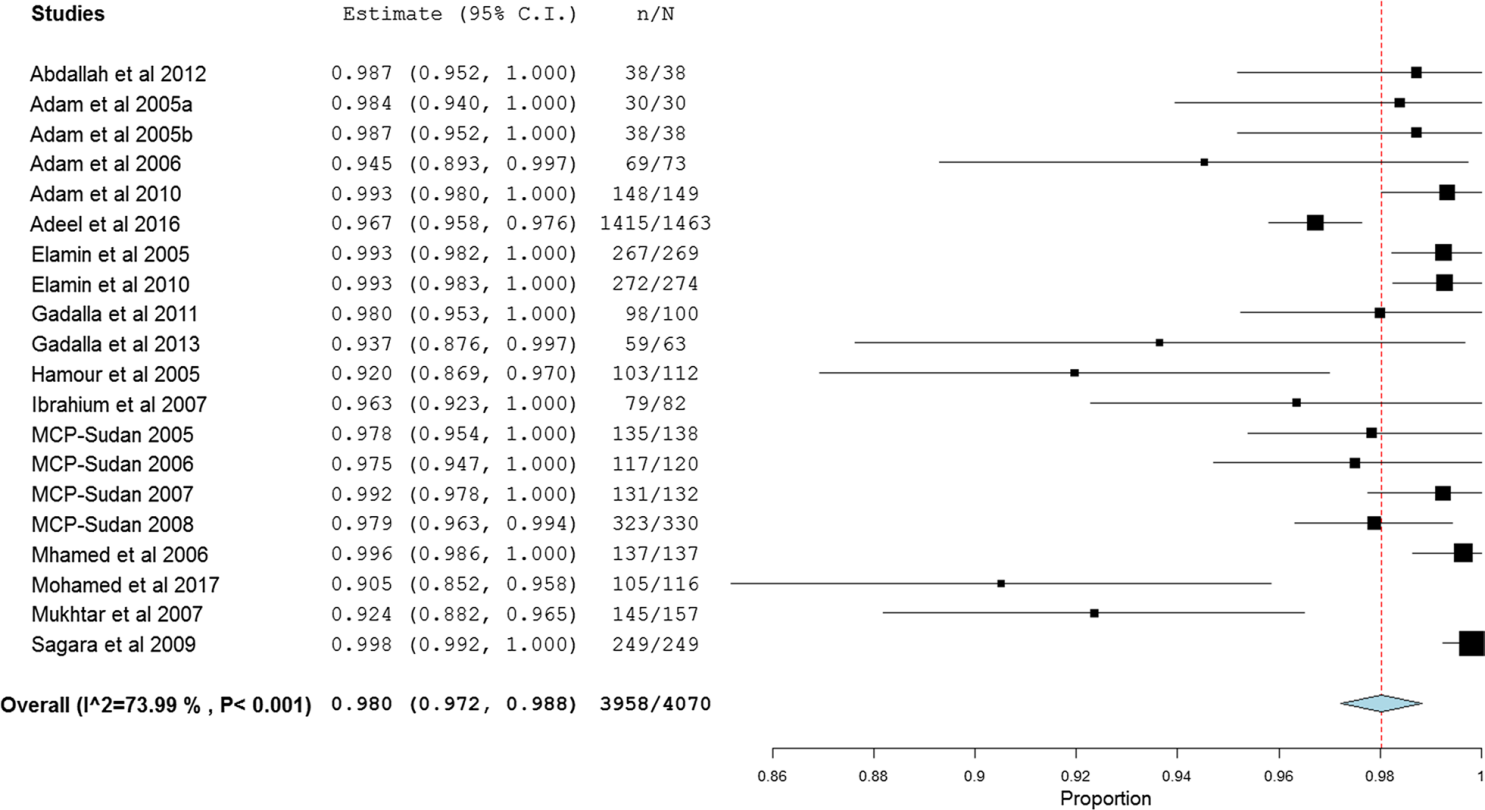
The overall treatment success of artemisinin-based combination therapy

**Fig. 3:**
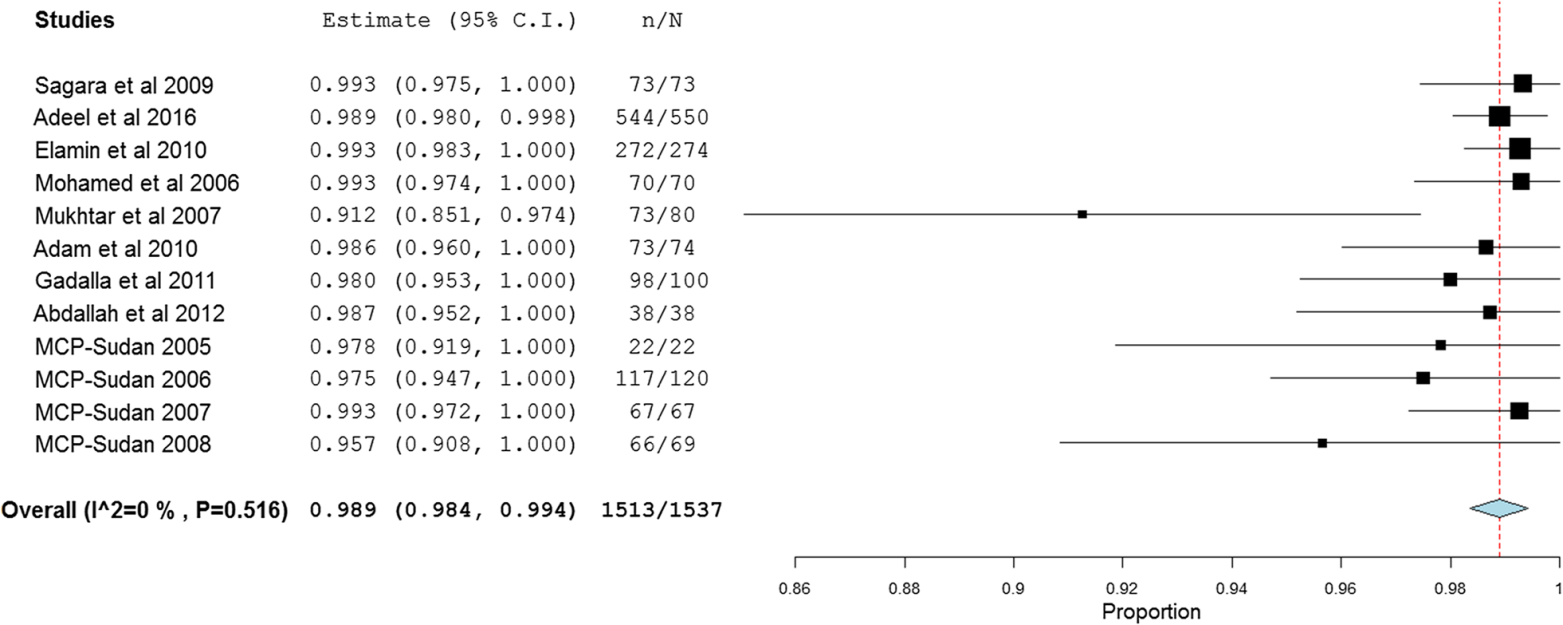
The overall treatment success of artemether + lumefantrine

**Fig. 4:**
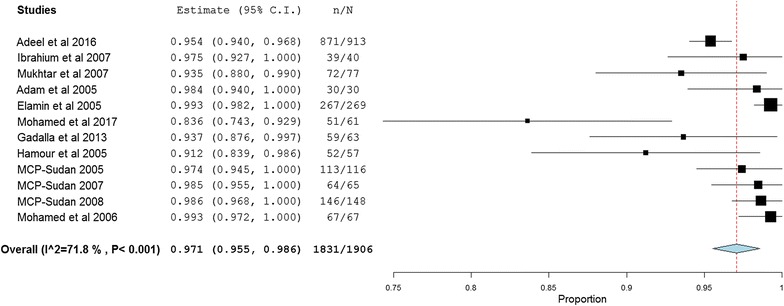
The overall treatment success of artesunate + sulfadoxine–pyrimethamine

Meta-analysis was conducted to investigate the overall treatment outcomes across the included studies in the different areas of Sudan. All the included 20 studies used polymerase chain reaction (PCR) genotyping and per protocol analyses. Overall, there was a significant high malaria treatment success (98% (95% CI 97.2–98.8%)) (Fig. 2). Seven studies showed 100% success rate (Additional file 3). Treatment with AL was found to have higher success rates compared to AS + SP (98.9% (95% CI 98.4–99.4%) vs 97.1% (95% CI 95.5–98.6%)) (Figs. 3, 4). Comparisons were also made based on the major different treatment regimens (the fixed dose of artesunate-sulfamethoxypyrazine-pyrimethamine (AS + SMP f) = 97.1% (90.5–100%), the loose dose of AS + SMP l = 99.2% (97.7–100%), artesunate-amodiaquine (AS + AQ) = 95.0% (90.2–99.8%), dihydroartemisinin-piperaquine (DHAP) = 99.1% (97.5–100%), artesunate + mefloquine (AS + MQ) = 94.74% (87.6–100%), and artemisinin and piperaquine (Artequick) = 98.23% (95.8–100%) (Additional file 4).

### Malaria treatment adverse drug reactions

Eleven (55.0%) out of the included studies (20) reported ADRs to ACT which were observed in 4.65% (184/3957) of the patients. All of the ADRs were mild (nausea, abdominal pain, diarrhoea, dizziness, rash) and resolved spontaneously. Five studies reported vomiting which was mild and did not require re-administration of the drug under study. The reported ADRs ranged from 2.63%, which were reported in AS + MQ, to 24.43%, which were observed in the fixed-dose 24-h regimen of AS + SMP f. There was no severe ADRs or deaths (Additional file 5).

## Discussion

The main finding of this meta-analysis is the high success rate (98%) of ACT in the treatment of uncomplicated falciparum malaria in Sudan. In neighbouring Ethiopia a high treatment success rate (92.9%) of malaria treatment was recently reported in a meta-analysis which included 21 studies with a total of 3123 patients. Furthermore, the treatment success was higher in falciparum malaria patients treated with AL compared to patients who received chloroquine for vivax malaria (98.1 vs 94.7%) [36]. In another recent meta-analyses in Ethiopia a high cure rate (98.2%) for AL was reported in 10 studies involving 1179 patients with uncomplicated falciparum malaria [37].

The current meta-analysis showed that the success rate of treatment with AL was higher compared to AS + SP (98.9 vs 97.1%). A high cure rate for AS + SP (98.0–100.0%) and a full efficacy (100%) have recently been reported in Yemen [38]. Interestingly, a recent clinical trial in Somalia reported a high (above 10%) treatment failure of first-line treatment (AS + SP), which is the threshold suggested by WHO to change malaria treatment policy [39]. In some parts of Somalia the failure rate of AS + SP was 22% [40]. The inadequate/inefficient health system in Somalia might be behind the high failure rate of AS + SP. However, a high success rate of AL was reported in Somalia [41]. It should be noted that Sudan is among the few African countries where AS + SP is first-line treatment for uncomplicated falciparum malaria. Few trials and reviews on AS + SP for the treatment of uncomplicated falciparum malaria are available in Africa and in these AS + SP was inferior even to non-ACT [42].

Because of fear of the spread of resistance to SP and the availability of the drug itself (AS + SP) in the international market, in 2017 Sudan changed the treatment of uncomplicated falciparum malaria to AL and DHAP as first- and second-lines of treatment [43]. The success rate of DHAP (2 studies) was 99.1% in this meta-analysis. This agrees with the findings of the recent Cochrane Database Systemic Review where DHAP reduced overall treatment failure compared with AL [41]. Likewise, the result of the former Cochrane Database Systemic Review (2009) showed that DHAP performance was superior to AS + MQ (in Asia) and AL in Africa [42].

The current meta-analysis showed that 11 studies reported ADRs to anti-malarial treatment (4.64%, 184/3957). The highest rate (24.43%) of ADRs was reported in the fixed-dose 24-h regimen of AS + SMP. The repeated dose of the fixed-dose 24-h regimen of AS + SMP f within short duration (24 h) rather than the 3-days regimen could explain the high rate of ADRs reported. The rate of ADRs was lower than the rate reported in the recent review in Ethiopia where 344 of 822 patients had ADRs, with a pooled event rate of 39.8% [40]. In all studies included in this review, there was no reported case of death due to malaria complications or due to ADRs. This might be explained by the fact that these studies were conducted among participants with uncomplicated malaria rather than the severe form which can lead to death.

One of the limitations of these studies was the poor recording of the ADRs. The other important limitation was lack of coverage of western Sudan. The vast majority of these studies were in eastern and central Sudan, perhaps due to the presence of large agricultural schemes in central and eastern Sudan and the endemicity of malaria.

## Conclusion

Based on this review, the overall malaria treatment success was high, with some regimens showing higher efficacy compared to others. Overall the regimens were associated with non-serious low rates of ADRs. Although the results of this review showed that the current malaria treatment agents in Sudan are effective and safe [44], greater efforts need be taken to develop new and more potent anti-malarial agents to prevent resistance which has been reported in other areas of the world.

## Additional files

**Additional file 1.** Characteristics of all studies of this systematic review and meta-analysis (included, excluded and unpublished studies).

**Additional file 2.** Assessment of publication bias.

**Additional file 3.** Characteristics of the included studies with 100% success rates.

**Additional file 4.** Comparisons of success rate based on different treatment regimens.

**Additional file 5.** Adverse drug reactions of anti-malarial treatment.
